# Comparative Analysis of Genome Diversity in Bullmastiff Dogs

**DOI:** 10.1371/journal.pone.0147941

**Published:** 2016-01-29

**Authors:** Sally-Anne Mortlock, Mehar S. Khatkar, Peter Williamson

**Affiliations:** Faculty of Veterinary Science, The University of Sydney, New South Wales, 2006, Australia; University of Florida, UNITED STATES

## Abstract

Management and preservation of genomic diversity in dog breeds is a major objective for maintaining health. The present study was undertaken to characterise genomic diversity in Bullmastiff dogs using both genealogical and molecular analysis. Genealogical analysis of diversity was conducted using a database consisting of 16,378 Bullmastiff pedigrees from year 1980 to 2013. Additionally, a total of 188 Bullmastiff dogs were genotyped using the 170,000 SNP Illumina CanineHD Beadchip. Genealogical parameters revealed a mean inbreeding coefficient of 0.047; 142 total founders (*f*); an effective number of founders (*f*_e_) of 79; an effective number of ancestors (*f*_a_) of 62; and an effective population size of the reference population of 41. Genetic diversity and the degree of genome-wide homogeneity within the breed were also investigated using molecular data. Multiple-locus heterozygosity (MLH) was equal to 0.206; runs of homozygosity (ROH) as proportion of the genome, averaged 16.44%; effective population size was 29.1, with an average inbreeding coefficient of 0.035, all estimated using SNP Data. Fine-scale population structure was analysed using NETVIEW, a population analysis pipeline. Visualisation of the high definition network captured relationships among individuals within and between subpopulations. Effects of unequal founder use, and ancestral inbreeding and selection, were evident. While current levels of Bullmastiff heterozygosity, inbreeding and homozygosity are not unusual, a relatively small effective population size indicates that a breeding strategy to reduce the inbreeding rate may be beneficial.

## Introduction

Modern breeds of dog have descended from the gray wolf, but through domestication and artificial selection, have diversified into a large collection of sub-populations, or breeds, with diverse morphological and physiological traits [1]. Since the canine genome was sequenced [2], there has been an increased interest in the genomic basis of canid variation, evolution and disease [3–5]. Concerns about the potential effects of inbreeding and reduced diversity on health and welfare within breeds, has also led to a call for improved genetic management practises [6–9]. Hence, managing diversity has become a major focus for dog breeders and oversight authorities [10]. National breed clubs are now assessing methods for evaluating genomic diversity to inform breeding decisions and reduce the incidence of disease, while maintaining positive breed traits and diversity.

Traditionally, genealogical data has been used to assess genetic diversity in dogs [11–15]. Commonly used descriptive parameters include, mean inbreeding coefficient, effective population size, and effective number of founders and ancestors [11]. Effective population size is the equivalent number of individuals contributing to the breeding population that would give rise to the observed variance in gene frequency and inbreeding rate in the population, the larger the effective population size the greater the predicted diversity [13, 16]. Inbreeding coefficient refers to the probability that alleles at a locus descended from a single allele from a common ancestor, a high coefficient indicating increased risk of inheriting deleterious alleles. However, the use of genealogical information is limited by incomplete or inaccurate pedigrees [17, 18]. As a result, molecular genotypes are a preferred source of data to analyse diversity in dogs and other domestic species [1, 12, 19, 20]. Canine high density single nucleotide polymorphism (SNP) genotyping tools, now provide a platform to generate molecular data for analysis. Molecular parameters, such as the level of multi-locus heterozygosity, inbreeding coefficient and effective population size, can be calculated to assess diversity. Multi-locus heterozygosity is a measure of genetic variability and is the proportion of heterozygous loci across an individual’s genome, higher heterozygosity associated with the ability to respond to selection pressures.

The level of diversity calculated using molecular methods may differ from those calculated using genealogical methods. Genealogical methods are more likely to pick up recent events that influence diversity, such as recent inbreeding and use of popular breeding individuals. However, molecular data reflects the cumulative effects of selection, migration and past breeding events that predate pedigree records.

Bullmastiffs are a large working breed originating in Britain in the mid 1800’s as estate guardians to ward off poachers [21]. They were bred by gamekeepers for strength, size and speed using a cross of the tough, heavy and aggressive Bulldog of the 19^th^ century with the large, strong, less aggressive Mastiff. The Bullmastiff breed was deemed pure by the Kennel Club in 1924, with a genetic background of approximately 60% Mastiff and 40% Bulldog [21]. Health concerns within the breed include hip and elbow dysplasia, progressive retinal atrophy, bloat, and cancer, with a relatively high incidence of lymphoma and mast cell tumours [22–24]. The history of the breed and the incidence of diseases that contribute to a shortened life-span, make this breed an interesting subject for analysis of diversity, genome function and pathophysiological traits [25].

The aim of this study was to evaluate genomic diversity and characterise the genetic structure of Bullmastiffs, using both pedigree and genome-wide molecular data. The results show that the breed has a relatively small effective population size and limited genetic diversity, but is similar to a number o fother breeds with similar population numbers. The knowledge gained from this study may be used to manage and preserve genetic diversity in Bullmastiffs by informing the development of breeding strategies, and will also be useful for disease association analysis in this breed.

## Methods

### Selection of dogs

A total of 16,739 registered Bullmastiff dogs were included in the genealogical analysis including 250 internationally bred dogs. Out of these, 188 Bullmastiff dogs belonging to over 40 different Australian kennels together with recent international imports and their direct decedents, aged from two months to nine years, were genotyped using genetic material isolated from blood and semen samples. Dogs genotyped were all owned pets and were volunteered with informed consent.

### Genealogical Analysis

Pedigree records of 16,739 Bullmastiffs registered between 1980 and 2013 were provided by Dogs NSW [26]. The genotyped dogs and their ancestors (n = 993) were used as a reference population, and were a subset of the 16,739 registered dogs. Analysis was performed using PEDIG software [27]. The average complete equivalent generations (EqG) and generation interval/length (*l*) were computed based on the reference population by PEDIG programs ngen and intgen, respectively. EqG is the total proportion of known ancestors at each generation over all generations [27], and generation interval is the average age of the parents when progeny are born. The following parameters were also calculated for the reference population using the PEDIG program, prob_orig: total number of founders (*f*), effective number of founders (*f*^e^) and effective number of ancestors (*f*^a^). *f*^e^ is defined as the number of equally contributing founders that account for the genetic diversity observed in the population, while *f*^a^ is the effective number of ancestors that account for this diversity, taken as the lower boundary of the marginally contributing ancestors [28]. The PEDIG program, meuw, was used to calculate the inbreeding coefficient for each dog in the total population and for genotyped dogs alone [29]. The mean inbreeding coefficients according to year of birth from 1980–2013 and inbreeding rate, were also calculated.

Effective population size (N_e_) was estimated from the rate of inbreeding per generation (ΔF) using N_e_ = 0.5ΔF in the program ENDOG v4.8 [28]. The relative increase in inbreeding by generation (ΔF) for the reference population was calculated using the following regression approach:

$$\Delta F=\frac{{\text{F}}_{n}\;-{\text{ F}}_{n-1}}{1-{\text{F}}_{n-1}}$$

where F_n_ − F_n−1_ = *l* × *b*, F_n_ being the mean inbreeding for the reference population, *b* the regression coefficient of the average inbreeding over year of birth, and *l* the average generation interval [28]. ENDOG computes the regression coefficient (*b*) of the inbreeding coefficient over the number of equivalent complete generations. Percentage contribution of ancestors to the genetic diversity was also calculated in ENDOG using an iterative procedure in which the marginal contribution of each ancestor is calculated [17].

### Molecular Analysis

#### DNA extraction

Whole blood was collected from 181 dogs using EDTA coated vacutainers. Genomic DNA was isolated from the blood samples using QIAamp DNA Blood Kit following the manufacturers protocol (QIAmp; Qiagen, Melbourne, Vic). Genomic DNA was also isolated from seven semen samples using a modified phenol/chloroform extraction procedure [30]. Frozen semen samples were thawed and 70μl aliquots were washed twice with PBS to remove any cryoprotectant. Each sample was incubated at room temperature for 1 hour with 100μl of sperm lysis buffer (1% 2-mercaptoethanol, 10mM Tris-HCl pH8, 100mM NaCl, 10mM EDTA pH8 and 5% SDS) followed by overnight incubation with 0.5 mg/ml proteinase K at 55°C with shaking. Following incubation, 100μl AE buffer (Qiagen, Melbourne, Vic) was added, followed by 100μl phenol and 100μl chloroform successively. The samples were vortexed and left at room temperature for 5 minutes. The mixture was spun at 10,000 rpm for 10 min before the upper aqueous layer was transferred to a new tube along with 200μl chloroform and 15μl of 4M NaCl, samples vortexed and left at room temp for 5 min. The mixture was spun again at 10,000 rpm for 10 min and the upper aqueous layer transferred to a new tube along with two equal volumes of 100% ethanol. The DNA fibre was spun down at 10,000 rpm for 10 min and the supernatant removed. The DNA pellet was washed with 70% ethanol then resuspended in 50μl AE elution buffer (Qiagen, Melbourne, Vic).

#### Genotyping

Genomic DNA was prepared for SNP analysis and genotyping was performed by centralised facilities of Geneseek, Lincoln, NE, USA using the CanineHD BeadChip (Illumina, San Diego, CA, USA). The high density bead chip provides comprehensive genome-wide coverage using over 170, 000 evenly spaced SNPS.

#### Multi-locus heterozygosity, homozygosity and inbreeding

Quality control filtering of the genotypic data was performed using PLINK v1.07 software [31]. The dataset was filtered to exclude individuals with >10% missing genotypes, to retain SNPs with a minor allele frequency (MAF) above 0.05, and SNPs with >90% genotyping rate. Multiple-locus heterozygosity (MLH), runs of homozygosity (ROH) and inbreeding coefficients were also computed in PLINK v1.07. MLH was calculated using Hardy-Weinberg test statistics and ROH within the genomes of the 188 individuals identified using the “–homozyg” command. The distribution of distances between SNPs was used to determine appropriate parameters to define a ROH. To exclude short and highly prevalent ROHs resulting from high levels of LD, the minimum length of a ROH was set to 1000kb. Using a method proposed by Purfield et al., [32] a minimum run length of 49 SNPs is required to produce <5% ROH that can occur randomly by chance. To account for 1% error in genotyping calls using the bead chip, up to 1 heterozygote was allowed in each run. The following parameters were used: homozyg-window-het 1, homozyg-snp 50, homozyg-window-snp 50, homozyg-window-missing 5, homozyg-window-threshold 0.05, homozyg-kb 1000, homozyg-density 50, homozyg-gap 100. Runs of homozygosity (ROH) then calculated as a proportion of the canine genome. ROH were used to calculate inbreeding using a formula devised by McQuillan, et al. [33] whereby the total length of ROHs covering an individual’s genome (L_ROH_) is divided by the length of autozomal genome covered in SNPs on the chip (L_AUTOSOME_); *F*_ROH_ = *L*_ROH_/*L*_AUTOSOME_. This was calculated using ROH lengths >1Mb (F_ROH>1Mb_), >2Mb (F_ROH>2Mb_), >4Mb (F_ROH>4Mb_) and >8Mb (F_ROH>8Mb_) corresponding to ROHs originating from ancestral populations 50, 25, 12 and 6 generations ago respectively [34]. Inbreeding coefficients for genotyped dogs were calculated based on the observed versus expected number of homozygotes using the “–het” command. A Pearson correlation was computed between individual genealogical and molecular inbreeding coefficients.

The level of heterozygosity and homozygosity in the Bullmastiff population was compared across multiple breeds using additional genotype data from 202 dogs across 12 diverse breeds obtained from the LUPA Dataset, available for download (http://dogs.genouest.org/SWEEP.dir/Supplemental.html) [35]. The 12 breeds represent a variety of breed types and histories; the Bernese Mountain Dog, English Bulldog and Rottweiler being mastiff-like breeds; Border terrier and Jack Russell terrier represent terrier breeds; the Cocker Spaniel represents a spaniel breed; the Greyhound is a sight hound breed; the Labrador Retriever a retriever breed; the Weimaraner a scent hound and other less commonly classified breeds the Poodle, Nova Scotia Duck Trolling Retriever and Doberman [1, 36]. Quality control filtering of all data was repeated using 154,385 common SNPs across both datasets. Heterozygosity and homozygosity were calculated for each breed using the same parameters mentioned previously.

#### Effective population size

The effective population size (N_e_) was estimated using the linkage disequilibrium method implemented by the program NeEstimator Version 2.01 [37]. To avoid bias from close linkage between loci, 10,000 evenly placed SNPs from across the genome were selected for inclusion in the N_e_ calculation. The genome was divided into 10,000 segments and the SNP with the highest MAF within each region was selected; rare alleles with a MAF < 0.05 were excluded.

#### Population structure

Fine-scale population structure was investigated and visualised using NETVIEW, a population analysis pipeline [38]. An unsupervised network clustering procedure, Super Paramagnetic Clustering (SPC), was used to create a fully connected population network in which individuals are clustered based on genetic distance. An identity by state (IBS) relationship matrix created in Plink was imported into SPC and run with K = 10 and a minimum cluster size of 2. The binary edge file produced with SPC was combined with a relationship matrix file in R [38, 39] to generate a weighted relationship matrix. The weighted relationship matrix was then converted into a GML file using the format conversion tool in Network Analysis Tools (NeAT) [40]. The clustering of individuals within the network then visualised in Cytoscape using an organic visualisation style [41]. The average relationship between all genotyped individuals and within each cluster of individuals was also calculated from pedigree data using ‘pedigree’ and ‘kinship2’ packages in R [42, 43].

#### Genetic Relationship between breeds

The genetic relationship between Australian Bullmastiffs and other breeds was investigated using genotype data from the Bullmastiff dogs and 456 dogs belonging to 30 breeds in the LUPA dataset[35]. A genetic distance matrix was created in Plink and used to construct a neighbour joining tree in R using the ‘ape’ package [44].

#### Ethics Statement

The study was approved by The University of Sydney Animal Ethics Committee under protocols 4949 and 2013/6013.

### Results

#### Genealogical results

**Fig 1:**
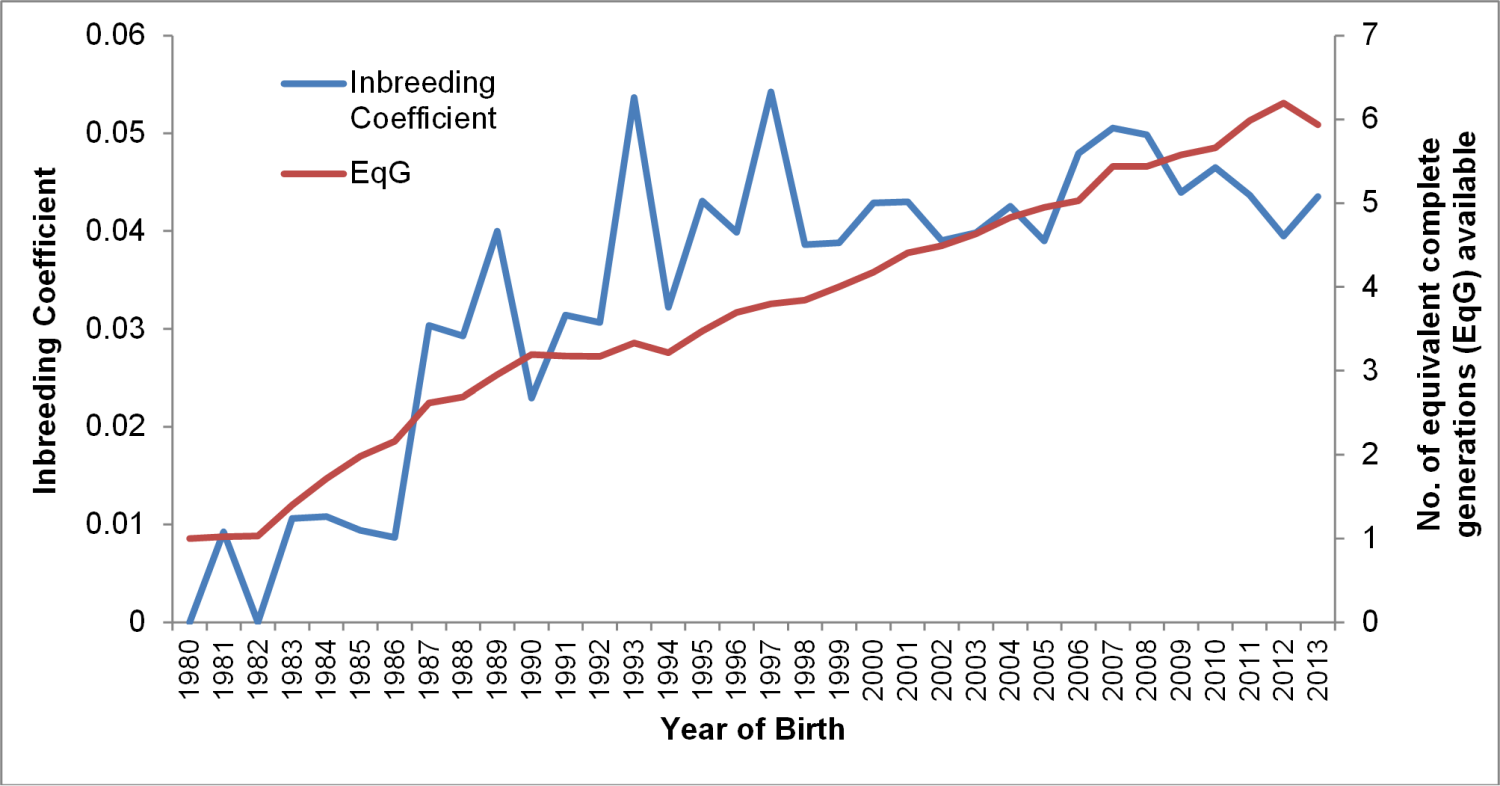
Inbreeding over time. Mean inbreeding coefficients and number of equivalent complete generations of Bullmastiffs by year of birth from 1980 to 2013.

A total of 16,739 Bullmastiff dogs were registered in Australia between 1980 and 2013, the number registered each year increased from 1980 to the mid 1990’s then stabilised, with an average of 655 dogs registered each year since that time (see S1 Fig). Genealogical parameters used to assess diversity are shown in Table 1. The mean inbreeding coefficient from these data was calculated to be 0.039, compared to a coefficient of 0.047 for the genotyped dogs alone. The number of complete equivalent generations, generation interval (*l*) and effective population size was estimated to be 3.24 ±0.18, 3.29 and 41, respectively. Number of total founders (*f*) (n = 142) was higher than the effective founders (*f*^e^) (n = 79); a *f*^e^/*f* ratio of 0.56, higher than the average ratio observed in breed registries of similar size (0.18)[13]. This is attributed to the unequal contribution of breeding individuals to future generations. The effective number of ancestors (*f*^a^) was calculated as 62, the ratio *f*^a^/*f*^e^ of 0.79 indicating a past bottleneck event, with 20 ancestors contributing more than 50% of the diversity in the population. Mean inbreeding coefficients were averaged over each birth year to show the trend of inbreeding over time, with an inbreeding rate of 1.2% per generation. Inbreeding coefficients ranged from 0 in 1980 to 0.054 in 1997, an overall increase in mean inbreeding coefficient is seen until the mid 1990’s, reaching 0.043 in 1995 and remain relatively stable to 0.044 in 2013, reflecting the trend seen in the number of registered dogs each year and in part the number of equivalent complete generations (Fig 1). Genealogical parameters for a range of diverse breeds estimated in previous studies are provided in S1 Table.

**Table 1:** Molecular and Genealogical measures of genetic diversity and homogeneity.

Measures of Diversity	Mean inbreeding coefficient (*F*)	Inbreeding rate per generation (ΔF)	Effective population size (*N*_e_)	Observed founders (*f*)	Effective founders (*f*_e_)	Effective ancestors (*f*_a_)	Mean multilocus heterozygosity (MLH)	Mean runs of homozygosity (% of genome)
Genealogical	0.047^a^ (0.039^b^)	1.2%	41	142^c^	79^c^	62^c^	N/A	N/A
Molecular	0.035	N/A	29.1	N/A	N/A	N/A	0.206	16.17

^a^ Calcuated using pedigree information from 188 genotyped dogs

^b^ Calculated using Bullmastiff database of 16 739 registered dogs

^c^ Calculated using pedigree data from reference population (993 dogs)

#### Molecular results

Following genotyping and frequency pruning 77,912 SNPs out of the original 172,939SNPs were removed from the analysis. Filtered SNPs included 3,365 SNPs that failed the call-rate threshold (geno > 0.1) and 74,607 that failed the minor allele frequency test (MAF < 0.05). Three individuals were removed for poor genotyping call rate (<73%) the remaining dogs had an average call rate of 98.45%. Molecular parameters used to assess diversity in Bullmastiffs are shown in Table 1. The proportion of the Bullmastiff genome covered with ROH, the average number of runs per dog, average number of SNPs in each run and inbreeding coefficients computed using ROH within four length categories (>1Mb, >2Mb, >4Mb, >8Mb) are shown in Table 2. The mean inbreeding coefficient, MLH and effective population size (N_e_) estimated from the 185 Bullmastiff dogs using molecular data was 0.033, 0.206 and 29.1, respectively. Similar distributions are observed across both molecular and genealogical estimates of inbreeding coefficients for genotyped dogs with a significant positive correlation between individual values (0.44, P < .001) (see S2 and S3 Figs). Mean inbreeding coefficients calculated for various other breeds using molecular data are provided in S2 Table. Genotype data from the 188 Bullmastiff dogs is available for download (https://osf.io/yzksh/).

**Table 2:** Proportion of the genome, runs of homozygosity inbreeding coefficient in Bullmastiff dogs.

Length (Mb)	Proportion of genome (%)	No. of runs	No. of SNPs per run	F_ROH_
>1	16.44	192.31	109.91	0.147
>2	9.51	75.32	164.82	0.081
>4	2.62	11.97	299.92	0.019
>8	0.35	0.80	579.18	0.002

In the multi-breed dataset, out of the 154,385 SNPs common to both the Bullmastiff and multi-breed populations, >80,000SNPs remained for each breed following genotyping and frequency pruning. Runs of homozygosity in these additional 12 breeds ranged from 6.08% in the Jack Russell Terrier to 14.69% in the British Bulldog (S3 Table). F_ROH>1Mb_ ranged from 0.061 in the Jack Russell Terrier to 0.151 in the Bulldog and F_ROH>8Mb_ ranging from 0 in the Doberman Pinscher and Bernese Mountain Dog to 0.006 in the Jack Russell Terrier. The mean MLH observed in Bullmastiff dogs was 0.200, compared with a range of between 0.179 and 0.286 across the 12 breeds (S4 Table).

**Fig 2:**
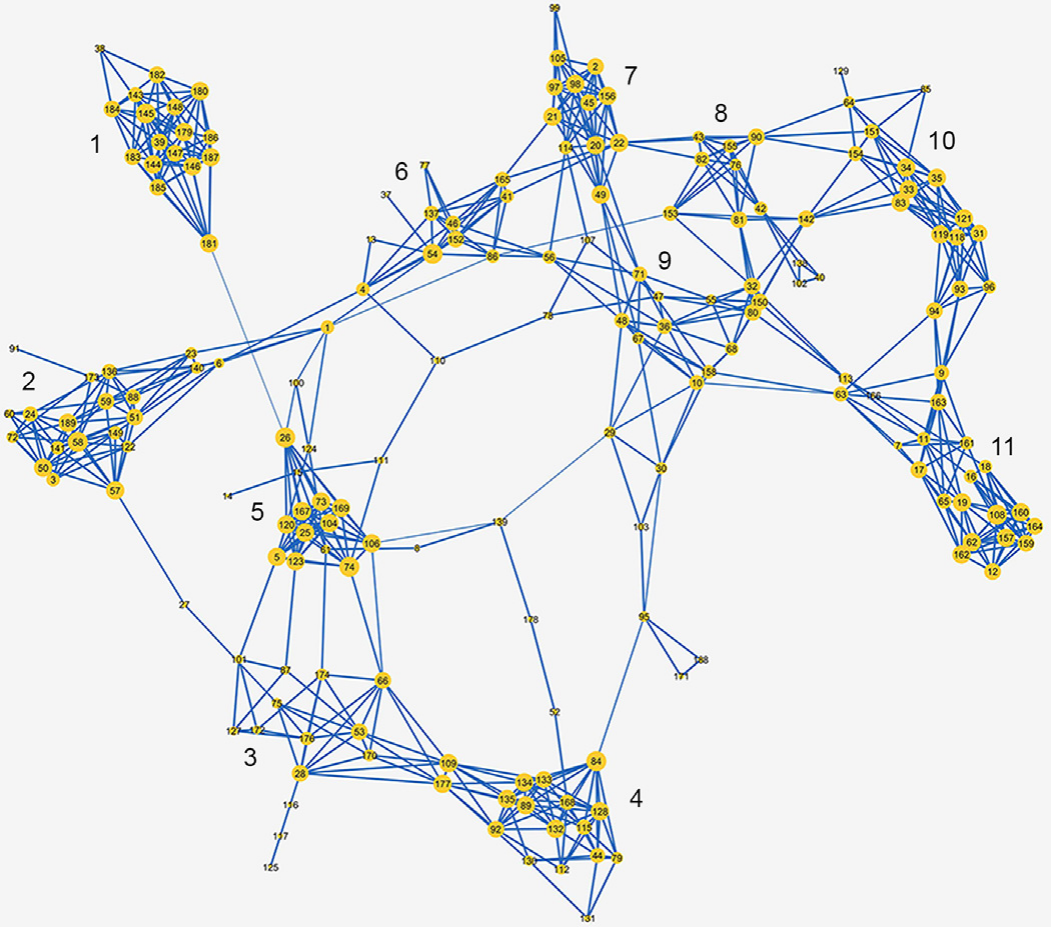
Genetic structure based on genotype data from Bullmastiff dogs. High-definition network visualisation of population structure in Cytoscape. More closely related individuals are co-located. Thickness of edges is proportional to genetic distance while node size varies in proportion to number of edges, showing degree of relatedness of individuals within the dogs. Subpopulations are labelled 1 to 11.

The molecular relationship between Bullmastiffs genotyped is depicted in the high-definition network visualisation (NETVIEW) diagram (Fig 2). The fine-scaled population structure revealed by NETVIEW highlights the presence of eleven main subpopulations identified as clusters, with individuals represented as nodes and co-located on the basis of relatedness. Edges connecting nodes correspond to the individual relationships between dogs. The node size reflects the number of edges and the thickness of edges inversely is proportional to genetic distance. The average relationship coefficient of all the dogs genotyped was calculated as 0.02 from pedigree data with an average of 0.18 within each of the clusters. The average relationship coefficient in each of the clusters labelled 1–11 in Fig 2 is presented in S5 Table.

**Fig 3:**
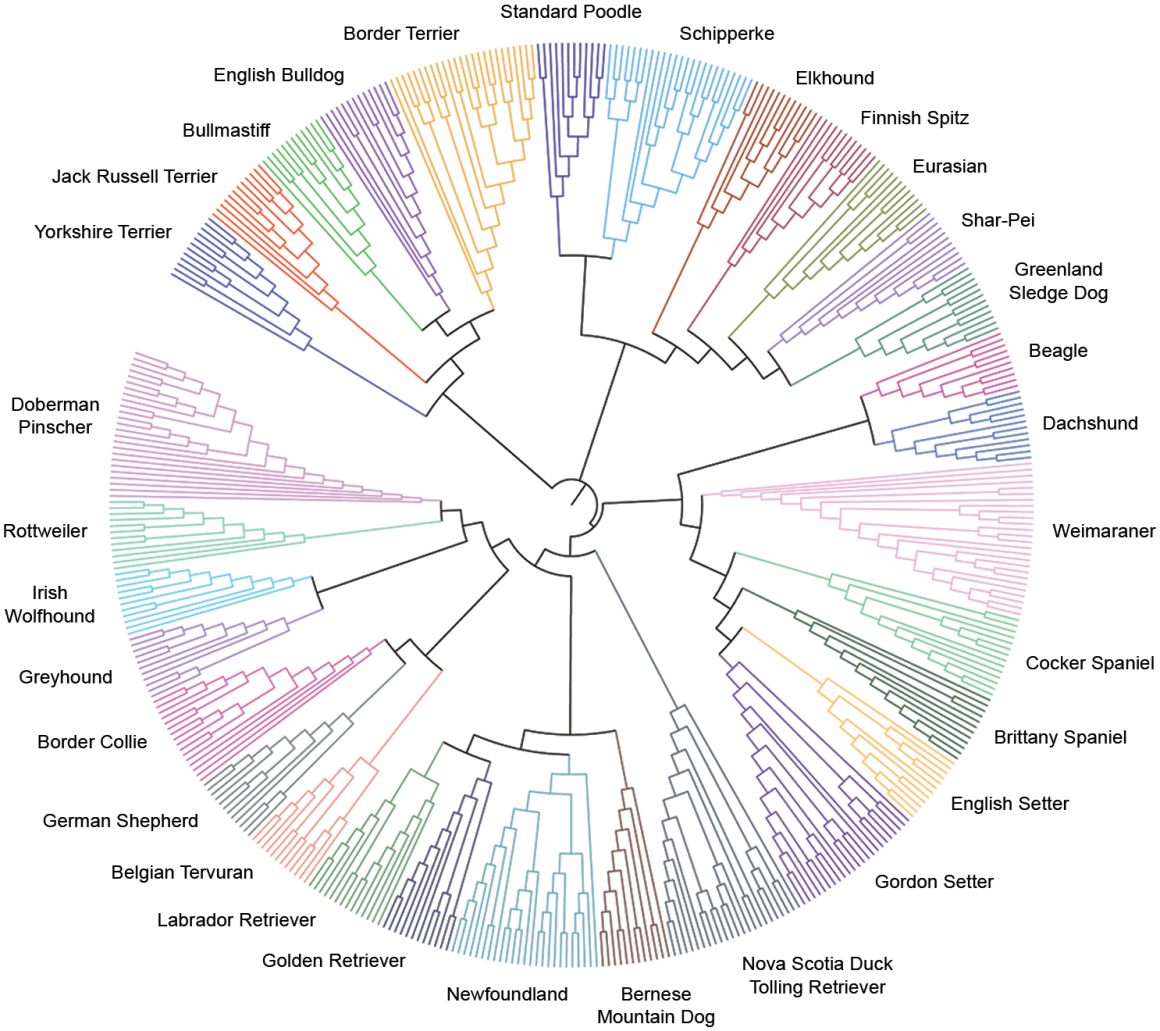
Neighbour-joining tree of 31 diverse dog breeds. Constructed using genetic distances calculated from >80,000 SNP loci genotyped. Each breed is represented with a unique colour.

The neighbour joining tree (Fig 3) constructed from genotype data from 12 Bullmastiffs and 456 dogs from the LUPA Dataset highlights the close genetic relationship between the Bullmastiff and the English Bulldog, one of its founding breeds. Distinct clusters within the neighbour joining tree correspond to different breeds within the multi-breed dataset. Breeds with similar phenotypic characteristics cluster closely, and include retrievers, scent hounds, sight hounds, terriers and spaniels, consistent with that seen in other studies [1, 35]. The limited internal structure of the tree, along with the short length of the internal branches, indicates that there is a strong genetic influence of bottlenecks during the creation of modern breeds which have diverged from a common population over a relatively short period of time. All dogs were correctly assigned to the breed from which they were sampled.

## Discussion

The aim of this study was to assess genetic diversity and population structure of Bullmastiff dogs using genealogical and molecular parameters as estimates. Genealogical parameters reflect more recent events while molecular parameters reflecting the cumulative effect of past drift, selection and migration [12]. The results reveal that Bullmastiffs generally have a level of genetic diversity that is mid-range when compared with other breeds, but a relatively low effectivepopulation size, and a rate of inbreeding that slightly greater than that the level required for avoiding the effects of inbreeding depression. This evaluation provides information that may influence decisions to maintain genetic diversity within the breed. Any decisions that affect the breed as a whole would be most effectively implemented through the collaborative efforts of breed clubs that maintain data records of registered dogs, and can provide information to their members. There are many examples where breed clubs have implemented registration policies and provided the best available advice to reduce the incidence of inherited diseases and deleterious alleles, and promote breed health.

Genealogical estimators of diversity rely on the completeness of pedigree data. EqG is the total proportion of known ancestors at each generation over all generations [27]. EqG is a measure of pedigree completeness, low EqG can be the result of recent breed establishment or incomplete pedigree records [28]. Previous studies in dogs have considered EqG values < 3 to be poor [12, 13]. The average number of complete equivalent generations present in the reference population was 3.24, providing sufficient depth for further computation of additional parameters.

The contribution of founders and ancestors to the genetic variation within the breed was evaluated through the estimation of total number of founders (*f*), *f*^e^ and *f*^a^. The *f*^e^ of 79 calculated in Bullmastiffs is much smaller than the total number of founders 142, with a *f*^e^/ *f* ratio of 0.56. A ratio closer to 1 indicates founders contributed equally to the subsequent generation; smaller ratios indicate fewer founding animals contributed to the observed genetic diversity [13]. The ratio of 0.56 for Bullmastiffs can be attributed to an unequal use of founders suggesting that some individuals have contributed to subsequent generations more than others [45]. Similar methods of calculating *f*^e^/ *f* using PEDIG software have been used in studies on other breeds with similar ratios found in Australian populations of Ibizan Hound and Sussex Spaniel and a European population of Barbets, a range between 0.05 and 0.82 observed across many domestic breeds (S1 Table) [13–15]. All four breeds have a relatively small registry size < 17,000. Founder contributions within larger registries (>30,000 registered dogs) in Australia are more variable. The unequal use of founders in the Bullmastiffs is less than that of other breeds with registries of similar size, and an average ratio of 0.18 [13].

The ratio of two parameters *f*^a^ / *f*^e^ can be used to identify any loss in genetic variability attributed to bottlenecks or an unbalanced use of sires and dams, with a ratio of 1 indicating a balanced use [17, 28]. Overuse of sires and dams in the population can lower the number of effective ancestors accounting for the genetic diversity in the population with more animals related to the top breeders. Similarly in the event of a bottleneck the number of breeding individuals in the population is reduced, causing the number of effective ancestors to drop along with the *f*^a^ / *f*^e^ ratio. This ratio has been calculated for a number of breeds ranging from 0.20 in Braque Saint-Germain to 0.94 in Border Collies [12, 46]. The ratio calculated in Bullmastiffs was 0.79, similar to that observed in Australian populations of German Shepherds and Smooth Collies and European populations of Bavarian Mountain Hounds, Boxers and Dobermans, all computed using similar methods (S1 Table) [12, 13, 46]. The registry size of these breeds varies, ranging from 1,500 in Smooth Collies to over 250,000 in German Shepherds, previous studies showing no obvious relationship between registry and population size. Instead *f*^a^ / *f*^e^ is a reflection of breeding management and bottleneck events as seen in European populations of Braque Saint-Germains and Hanoverian hounds, both having experienced a bottleneck with ratios of 0.20 and 0.48 respectively [12, 46]. The average ratio in breeds with similar registry size to Bullmastiffs in Australia is 0.47 [13]. Many breed populations in Australia have been affected by bottleneck events during introduction resulting in a *f*^a^ / *f*^e^ ratio of around 0.5 regardless of registry size [13]. The ratio of 0.79 indicates that Bullmastiffs have not been as severely affected by a bottleneck event or unequal sire use in comparison to other breed populations, but there is still evidence of unequal contributions from breeding animals that could be addressed by providing information and advice on breeding strategies [12, 13].

Monitoring the degree of inbreeding in a population is important to ensure that the rate of inbreeding is not excessive. Maintaining maximum diversity reduces the frequency of deleterious alleles in the population and reduces the incidence of heritable diseases. A previous study investigating genealogical parameters for a number of breeds within Australia found that the mean inbreeding coefficient ranged from 0 to 0.101 across 32 breeds investigated [13]. The results of the present study show possible limitations of using genealogical data alone. Whilst there was a positive correlation between individual estimates of inbreeding calculated using genealogical and molecular methods, the average inbreeding coefficient differs between methods, likely due to incorrect and incomplete pedigree information. The ability to identify distant founding animals and their co-ancestry is limited by the depth of pedigree data available, genealogical methods are therefore unable to reflect the true levels of identical by decent homozygosity present in the population [47]. Lack of pedigree information on imported dogs entered into local databases can also lead to inaccurate estimation of inbreeding and relationship coefficients. An inbreeding coefficient of 0.047 was calculated from pedigree data available for the reference population, larger than the 0.035 value calculated using molecular data. The molecular estimate of inbreeding was similar to that estimated using the full pedigree database (0.039) suggesting molecular methods are able to capture values representative of the broader population. These values are within the range seen in large registries, including the Labrador Retriever, Border Collie, Cavalier King Charles Spaniel and some smaller registries including the Dachshund, Samoyed and Polish Lowland Sheepdog (S2 Table) [12, 13, 48].

Inbreeding coefficients calculated using genealogical methods are largely dependent on the amount of pedigree information available. Therefore, the rate of inbreeding per generation, ΔF, is also used as a measure of inbreeding in a population, and negative effects of inbreeding are usually a result of the rate at which inbreeding increases over time [11]. An inbreeding rate lower than 1% per generation is acceptable to limit the effects of inbreeding depression. The value for the Bullmastiffs fell outside this range, with a ΔF of 1.2% [11, 49, 50]. However, the mean inbreeding coefficient increased from 1980 to 1995 after which it stabilised. This trend reflects the equivalent complete generations available each year and the increase of registered dog numbers to 1995. The trend in inbreeding coefficients is very similar to those observed in a recent Bullmastiff breed analysis reported by Lewis et al., 2015 [51]. Similar patterns have also been observed in other breeds, with inbreeding coefficients increasing during establishment, and prior to importation of dogs and semen for breeding, or other changes to selective breeding [13, 51]. The rate of inbreeding is determined by the effective population size, while inbreeding levels appear to have stabilised over recent years the undesirable ΔF is the result of the small effective population size of the breed. Similar ΔF values have being observed in the Skye Terrier, Nova Scotia Duck Tolling Retriever and Fox terrier populations in Australia, all with small effective population sizes ranging from 40–47 (S1 Table) [13].

Effective population size (N_e_) is a measure of the long-term performance of a population, and explains the extent and pattern of genetic variation in a population. The N_e_ is important because it is a measure of the available genetic diversity within the breed. As the N_e_ decreases, there is an increased risk of mating genetically related animals. The N_e_ of 41 computed using pedigree information in the present study, is relatively low when compared to that calculated in other breeds. Studies using a similar method of calculating N_e_ have computed values ranging from 20 in the Barbet to 2136 in the West Highland White Terrier, which have small and large registered populations, respectively [12–15]. This reflects the tendency for breeds with large population sizes to also exhibit large effective population sizes [12–15], although this relationship can be markedly affected by genetic bottlenecks. The N_e_ seen in Bullmastiffs is similar to that found in a European populations of Romagna Water Dog and Irish Red Setter and Australian populations of Fox Terrier and Skye Terrier, all of which have small to medium sized registries below 20,000 dogs, with the exception of the Irish Red Setter which has a larger population [12, 13]. All four breeds have a low number of effective ancestors in comparison to effective number of founders suggesting the breeds have experienced a bottleneck or unequal use of breeding animals [12–14, 46]. The extent and distribution of genetic variation in a population can also be directly quantified using genetic markers. In this study genome-wide SNP genotype data was used to estimate the effective population size using a linkage disequilibrium (LD)-based method [37, 52]. Using LD, an N_e_ of 29.8 was estimated for Bullmastiffs, which was smaller than that found using pedigree data, and may be attributed to the level of pedigree completeness. A higher Ne of 101.5 has been estimated in a UK Bullmastiff population using the rate of inbreeding from 1980–2014, however the median value over five year intervals across this period was only 29.2 very similar to the Ne calculated in the genotyped population [53]. A population with N_e_ under 50 can be considered as more at risk from effects of inbreeding depression [49]. A small N_e_ is a reflection of the Bullmastiff population size, unequal use of breeding animals and an indication of unequal founder contributions, which is also suggested by the *f*^e^/*f* ratio.

Overuse of popular sires and unequal use of breeding individuals has a negative impact on effective population size and the dissemination of heritable diseases [54]. As only half the total numbers of founders account for the genetic diversity in the Bullmastiff population, and 20 ancestors account for over 50% of the diversity present, it is important for breeders restrict the overuse of individual breeding animals. Extensive use of popular sires was also observed in the UK Bullmastiff population, and has been a major contributor to a relatively high ΔF [51]. The current rate of inbreeding in the population may be contributing to an increase in the frequency of deleterious alleles in the population, and efforts to reduce the ΔF, especially within subpopulations where there is a high degree of relatedness between individuals, should be encouraged. Reduction in inbreeding rate will lead to an increase in effective population size. Simulations in a population of Dutch Golden Retrievers has shown restricting number of litters per sire and restricting use of animals with high relatedness to rest of population can decrease inbreeding rate [55]. Limiting the use of a sire to no more than 5 litters per year in a population of 150 breeding sires and 600 breeding females was more effective at reducing inbreeding rate than a whole life restriction [55]. Whole life restrictions led to a decrease in generation interval and faster replacement of the top sire with their sons, leading to an increase in inbreeding rate over time [55]. As the simulation was based on a population much larger than that of Bullmastiffs, a lower limit on litters per sire per year would be more effective at reducing inbreeding rate. Monitoring and limiting the genetic contribution of breeding individuals and their relatives has the potential to lower ΔF and improve N_e_.

Heterozygosity in Bullmastiffs is mid-range (MLH = 0.206) when compared to that calculated in other breeds using SNP data (range = 0.1 to 0.36 in modern breeds) [1, 2, 19]. This reflects the potentially available diversity within Bullmastiffs. The higher MLH observed in Bullmastiffs compared to breeds such as the Doberman Pinscher and British Bulldog may be attributed to the recent formation of the breed from the cross of two existing breeds. The lower MLH in Bulldogs may also reflect a severe bottleneck event in the mid 19^th^ century while Doberman pinschers underwent heavy selection in the late 19^th^ century, both events likely to have reduced the level of heterozygosity in the population [36]. Higher levels of heterozygosity observed in the Poodle and Labrador Retriever are likely to be the result of their large population sizes worldwide, and the availability of a large gene pool. The lower MLH may also be partly due the ascertainment bias of SNPs used for genotyping, and resulting from the SNP discovery process, whereby a relatively small number of dogs from major breeds have been used in the discovery panel [35]. However, we have applied stringent filters on the selection of SNPs for this analysis to minimise any effect of ascertainment bias.

Runs of homozygosity are contiguous segments of homozygous DNA sequence commonly produced by two mechanisms, a high degree of relatedness between individuals in a population, or positive selection [56]. A high level of relatedness can result from recent inbreeding, overuse of sires, past inbreeding or bottlenecks, all of which increase the likelihood of haplotypes being identical by decent [34, 56]. A variant producing a selective advantage will increase the frequency of the haplotype carrying that variant within a population, leading to a reduction in haplotype diversity [56]. Identifying the degree of homozygosity present in the population measures the effect of inbreeding on the genome, and provides information on the history of the population. The greater the proportion of the genome that is identical by decent, the higher the risk that deleterious recessive alleles will be inherited from both parents. Longer runs are derived from a recent ancestor and recent inbreeding; runs >8Mb corresponding to a common ancestor occurring only six generations ago (F_ROH>8Mb_), while shorter runs are derived from more distant ancestors and ancestral inbreeding; runs at least 1Mb in size corresponding to a common ancestor occurring 50 generations ago (F_ROH>1Mb_) [34]. Studies in humans and cattle have found that the proportion of ROH within a genome is a good, if not better estimate of individual autozygosity than traditional pedigree methods [33, 34]. A relationship has been identified between the number and length of ROH and inbreeding coefficients calculated from pedigree data, the length of ROH increasing with increasing inbreeding coefficients [57].

An average of 16.44% of the Bullmastiff genome was found to be covered by ROH > 1Mb, but only 0.35% of the genome was found to be covered by runs over 8Mb. The greater level of autozygosity estimated from ROH>1Mb compared to >8Mb in Bullmastiffs suggests that inbreeding and selection likely occurred during establishment of the breed, within the small ancestral population. This is supported by F_ROH_ estimates, an inbreeding coefficient of 0.15 corresponding to ROH>1Mb and a common ancestor 50 generations in the past, much larger then that calculated from larger runs F_ROH_>8Mb = 0.002 representing a common ancestor only 3 generations past. Whilst the small proportion of ROH>8Mb suggest inbreeding levels have improved the high proportion of the genome that is homozygous means they are at risk of inheriting two copies of deleterious recessive alleles.

A combined molecular genotyping dataset consisting of Bullmastiffs and an additional 12 breeds from the LUPA Dataset [35] allowed the parameters measuring diversity to be compared between breeds. Using common SNPs between datasets, an average of 14.10% of the Bullmastiff genome was found to be covered by ROH > 1Mb, the second highest out of the 13 breeds, but only 0.19% of the genome was found to be covered by runs over 8Mb. The greater level of autozygosity estimated from ROH>1Mb compared to >8Mb in Bullmastiffs suggests that inbreeding and selection was most likely to have occurred during establishment of the breed, and within the small ancestral population. This is supported by F_ROH_ estimates, an inbreeding coefficient of 0.15 corresponding to ROH>1Mb and a common ancestor 50 generations in the past is much larger then that calculated from larger runs F_ROH_>8Mb = 0.002 representing a common ancestor only 3 generations past. Breeds such as the Rottweiler and Jack Russel Terrier had lower levels of autozygosity >1Mb and smaller F_ROH>1Mb_ suggesting less effect of ancestral inbreeding possibly due to large effective population of the Rottweiler and wide out crossing during the establishment of the Jack Russel breed [12]. Jack Russell Terriers, Standard Poodles, and Labrador Retrievers, all have a higher proportion of their genome covered in longer runs >8Mb, and larger F_ROH>8Mb_ which could be attributed to more recent inbreeding or selection events. The standard poodle underwent a major bottleneck during the mid 20^th^ century which was associated with the overuse of show-winning bloodlines [58]. The small sample numbers from each breed in the LUPA Dataset present a limitation to the study as the relationships between these individuals in the same breed and how they represent the overall populations of these breeds is unknown.

The measure of genetic relatedness between Bullmastiff dogs was conducted using over 75, 000 data points and summarized into a complex relationship network. Visualisation of this data allows the genetic relationships between individual dogs and subpopulations within a breed to be easily identified and interpreted. The NETVIEW pipeline consists of a network-based clustering procedure (SPC) which infers high resolution population structure based on a genetic distance matrix which, when combined with network visualisation tools, produces a high-definition network visualisation of the population structure. When compared to other methods, such as Principle Component Analysis or Admixture, NETVIEW can reveal hierarchical structure and information on the relatedness of individuals within their clusters that the other methods fail to correctly identify [38]. It has the ability to detect previously unidentified fine-scale population substructure and genetic relatedness between individuals [38]. Evaluation of pedigree information revealed that eleven genetically distinguishable clusters identified in NETVIEW correspond to close family groups and pedigree lines with an average relationship coefficient within clusters of 0.18, higher than that of the genotyped population as a whole. Use of popular sires and dams within subpopulations can contribute to the close genetic relationship within clusters, many dogs within clusters sharing recent common ancestors and originating from the same kennels. Outlier dogs 116, 117 and 125 were recent UK imports and thus have fewer connections with the remaining population. Identification of these subpopulations highlights the different contributions of groups of individuals and pedigree lines to the genetic diversity of the breed. The importation of dogs and semen from the United Kingdom and USA is reflected in the population structure. Dogs with recent UK ancestors and bred from UK dogs cluster separately (clusters 3, 2 and 5) to dogs with recent USA ancestors (clusters 8 and 10), or lines with little recent international contributions. While imported dogs and their immediate descendants tend to cluster separately, they remain within close proximity to neighbouring clusters of primarily Australian bred dogs, highlighting the genetic similarity between Bullmastiff populations worldwide.

The neighbour joining tree presented in Fig 3 reflects the strong genetic relationship between the British Bulldog and the Australian Bullmastiff population, and is consistent with the role of the Bulldog in the creation of the Bullmastiff breed. The population structure, N_e_ and number of founding dogs reflect the history of the Bullmastiff in Australia. The Australian Bullmastiff population was primarily founded by UK dogs; the first Bullmastiff was introduced from Britain in 1949 with 57 dogs imported from the UK between 1949 and 1990. The geographic range of imports broadened in the 1990’s, dogs imported from the USA and European countries such as Finland and Norway along with semen samples from dogs around the world [59].

## Conclusions

Evaluation of the genetic diversity and structure of dog breeds may be used to inform and advise those responsible for developing breeding strategies that avoid or reduce the rate of inbreeding, and thus the frequency of deleterious alleles and inherited diseases. Generally, these strategies would promote the limited use of sires, and avoid close relative matings and extend the use of animals use din breeding. Both molecular and genealogical measures of diversity provided a comprehensive evaluation of the genetic diversity within Bullmastiff dogs. The molecular analysis overcomes limitations in calculations based on pedigree data, and takes into account recent breeding events plus the effects of past inbreeding, selection and genetic drift. Molecular data has the advantage of accurately measuring genetic heterogeneity between individuals used for breeding. Overall, the results of the present study show evidence of ancestral inbreeding in Bullmastiffs, and unequal founder contributions during breed establishment. A relatively small effective population size may be improved by utilising the available genetic diversity in systematic manner.

## Supporting Information

S1 Fig Bullmastiff Registrations. Number of Bullmastiffs registered in Australia each year from 1980 to 2013.
(TIF)

S2 Fig Distribution of inbreeding coefficients. The distribution of inbreeding coefficients across genotyped dogs calculated using molecular and genealogical methods.
(TIF)

S3 Fig Scatter plot of inbreeding coefficients. A scatter plot showing the relationship between inbreeding coefficients for individual dogs genotyped calculated using molecular and genealogical methods.
(TIF)

S1 Table Genealogical measures of genetic diversity in the Bullmastiff and 46 additional dog breeds. Values presented in this table are from previously published data estimated using similar genealogical methods to those in this study.
(PDF)

S2 Table Inbreeding coefficients calculated using molecular data for 24 dog breeds. Values previously published in [12].
(PDF)

S3 Table Proportion of the genome, runs of homozygosity and inbreeding coefficient in 13 dog breeds. (PDF)

S4 Table The average multilocus heterozygosity in 13 dog breeds. (PDF)

S5 Table Average relationship between genotyped dogs within clusters calculated using pedigree data. (PDF)
