# Supplementary figures and images for: Comparative Analysis of Genome Diversity in Bullmastiff Dogs
Source: PLoS One. 2016 Jan 29;11(1):e0147941. doi: 10.1371/journal.pone.0147941 (PMC4732815; doi:10.1371/journal.pone.0147941)

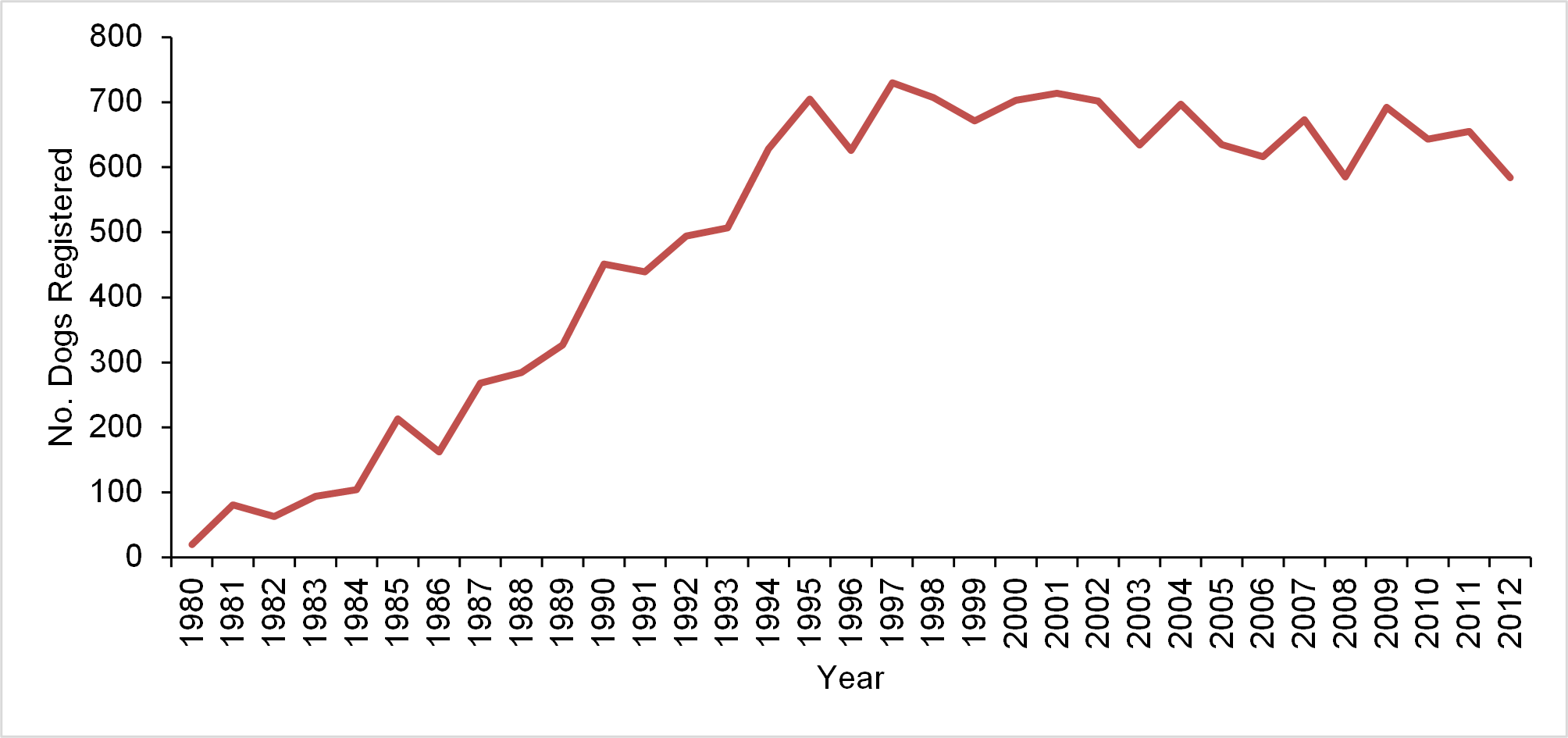

Supplement: S1 Fig — Number of Bullmastiffs registered in Australia each year from 1980 to 2013. (TIF) [file pone.0147941.s001.tif]

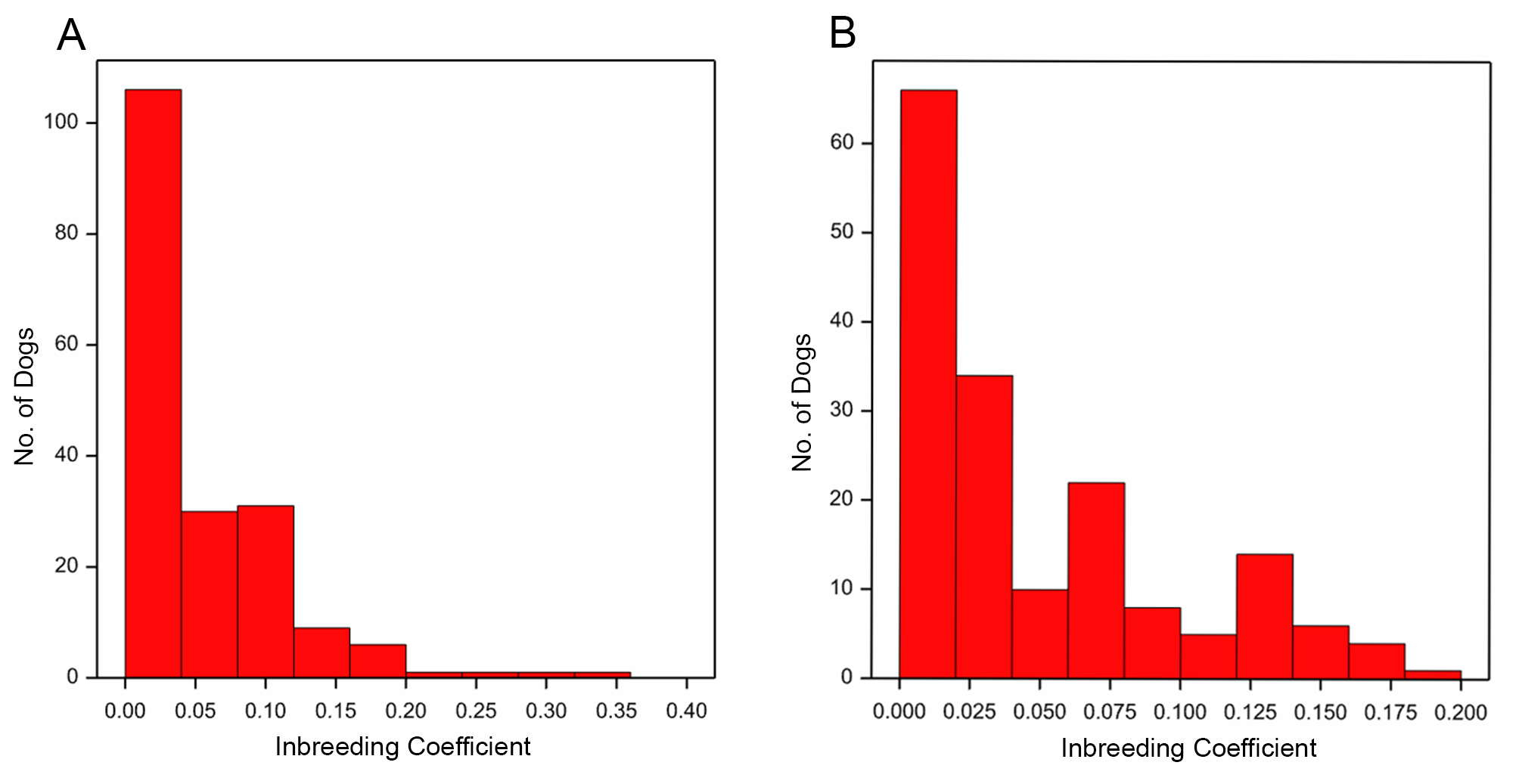

Supplement: S2 Fig — The distribution of inbreeding coefficients across genotyped dogs calculated using molecular and genealogical methods. (TIF) [file pone.0147941.s002.tif]

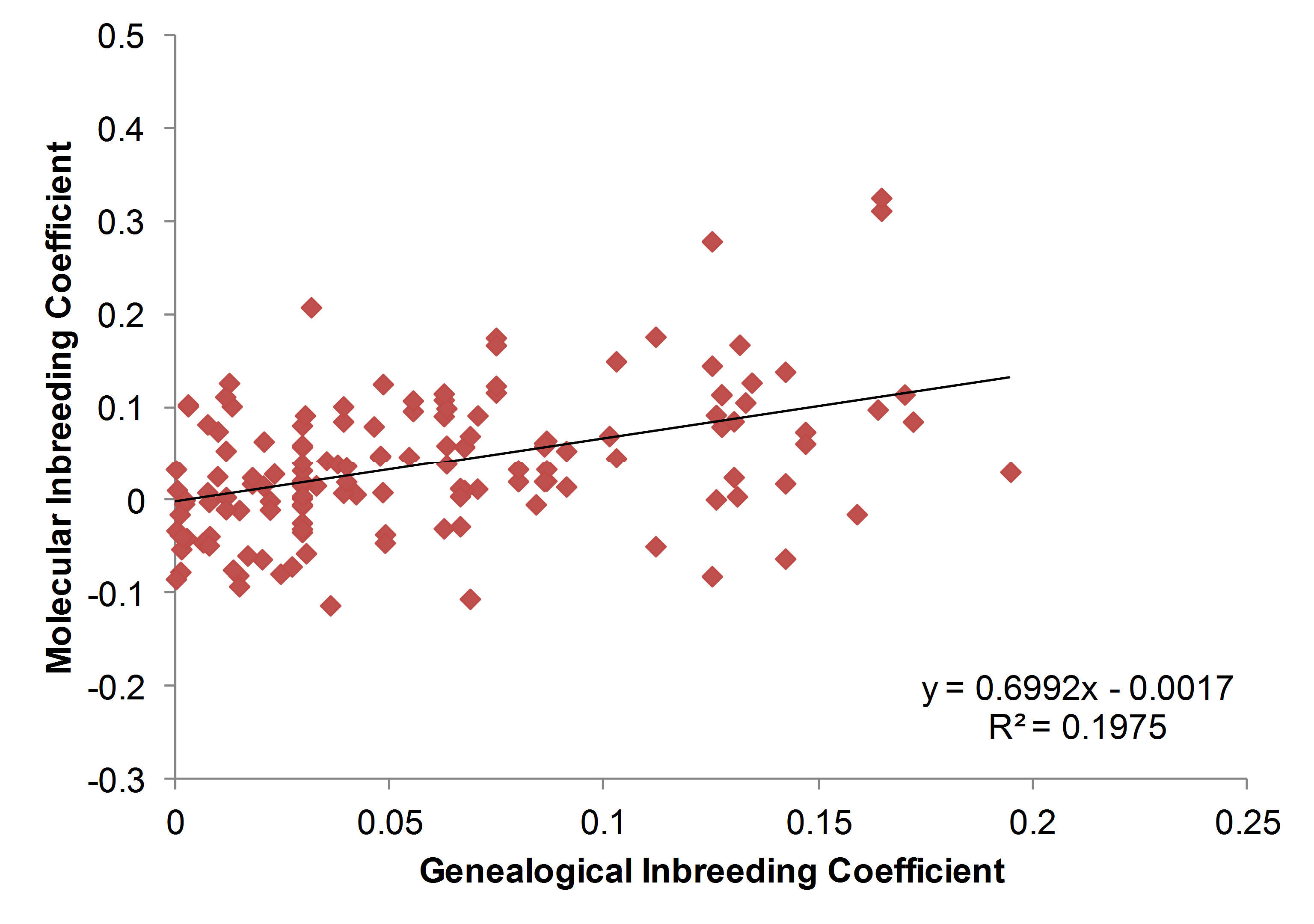

Supplement: S3 Fig — A scatter plot showing the relationship between inbreeding coefficients for individual dogs genotyped calculated using molecular and genealogical methods. (TIF) [file pone.0147941.s003.tif]
