# Supplementary material for: Comparative Analysis of Genome Diversity in Bullmastiff Dogs
Source: PLoS One. 2016 Jan 29;11(1):e0147941. doi: 10.1371/journal.pone.0147941 (PMC4732815; doi:10.1371/journal.pone.0147941)
Supplement: S1 Table — Values presented in this table are from previously published data estimated using similar genealogical methods to those in this study. (PDF) [file pone.0147941.s004.pdf]

S1 Table

Breed	Effective population size (N_e)	Mean inbreeding coefficient (*F*)	Ratio of effective no. of founders to total no. of founders (f_e/f)	Ratio of effective no. of ancestors to effective no. of founders (f_a/f_e)	Inbreeding rate per generation (ΔF)	References
Australian Cattle Dog	57.00	0.057	0.16	0.68	0.90	[13]
Barbet		0.064		0.90		[12]
	20.00	0.124	0.53	0.97		[14]
Bavarian Mountain Hounds	72.69	0.045	0.12	0.78	0.69	[47]
Beagle	76.00	0.046		0.27		[12]
	54.93				0.91	[45]
Bearded Collie	60.00	0.044		0.33		[12]
	23.91				2.09	[45]
Bichon Fries	53.00	0.101	0.20	0.48	1.00	[13]
	80.24				0.62	[45]
Border Collie		0.008		0.95		[12]
	129.00	0.041	0.17	0.60	0.40	[13]
	113.54				0.44	[45]
Boston Terrier	36.78				1.36	[45]
Boxer	113.00	0.043	0.19	0.54	0.40	[13]
	80.93				0.62	[45]
Braque Saint-Germain	29.00	0.075		0.20		[12]
	40.00	0.060	0.42	0.63		[14]
British Bulldog	101.00	0.056	0.24	0.37	0.50	[13]
Bull Terrier	295.00	0.011		0.40		[12]
	41.86				1.20	[45]
Bulldog	1216.00	0.013		0.30		[12]
	67.85				0.74	[45]
Bullmastiff	41.00	0.039	0.56	0.79	1.20	
Cavalier King Charles Spaniel	150.00	0.033		0.31		[12]
	204.00	0.035	0.31	0.44	0.20	[13]
	111.16				0.45	[45]
Collie (Smooth)	88.00	0.026	0.36	0.75	0.60	[13]
	90.03				0.56	[45]
Dachshund (Smooth-Haired)	59.07				0.85	[45]

Dachshund (Miniature Wire-Haired)	84.00	0.048	0.31	0.42	0.60	[13]
	110.39				0.45	[45]
Dalmatian	120.00	0.024		0.32		[12]
	142.41				0.35	[45]
Dobermann	187.00	0.023		0.72		[12]
	133.40				0.38	[45]
English Setter	195.00	0.021		0.31		[12]
	29.79				1.68	[45]
Fox Terrier (Smooth)	40.00	0.079	0.06	0.63	1.20	[13]
	80.40				0.62	[45]
German Shepherd Dog		0.018		0.85		[12]
	250.00	0.012	0.05	0.78	0.20	[13]
	147.51				0.34	[45]
Golden Retriever	219.00	0.013		0.44		[12]
	1090.00	0.051	0.15	0.56	0.00	[13]
	61.32				0.82	[45]
Great Dane	375.00	0.044		0.40		[12]
	166.77				0.30	[45]
Ibizan Hound	26.00	0.098	0.53	0.50	0.02	[13]
Irish Red And White Setter	46.00	0.058		0.23		[12]
	39.41				1.27	[45]
King Charles Spaniel	218.00	0.028		0.26		[12]
	41.64				1.20	[45]
Labrador Retriever	122.00	0.022		0.28		[12]
	153.00	0.034	0.13	0.50	0.30	[13]
	81.75				0.61	[45]
Lancashire Heeler		0.100	0.21	0.89		[15]
	32.78				1.52	[45]
Leonberger	722.00	0.028		0.52		[12]
	508.00	0.002	0.42	0.27	0.10	[13]
Manchester Terrier	23.83				2.10	[45]
Mastiff	70.55				0.71	[45]
Nova Scotia Duck Tolling Retriever	47.00	0.029	0.31	0.46	1.10	[13]
		0.260	0.52	0.53		[15]

Polish Lowland Sheepdog	69.00	0.039	0.82	0.71	0.70	[13]
Pug	133.88				0.37	[45]
Romagna Water Dog		0.003		0.42		[12]
Rottweiler	274.00	0.017		0.66		[12]
	191.00	0.025	0.22	0.55	0.30	[13]
	190.82				0.26	[45]
Samoyed	60.00	0.048	0.20	0.42	0.80	[13]
	64.72				0.77	[45]
Skye Terrier	40.00	0.061	0.49	0.37	1.30	[13]
Smooth-haired Dachshund	82.00	0.050		0.32		[12]
Staffordshire Bull Terrier	102.00	0.052	0.13	0.58	0.50	[13]
	97.71				0.51	[45]
Sussex Spaniel	55.00	0.023	0.52	0.67	0.90	[13]
Tyrolean hounds	26.48	0.095	0.19	0.65	1.88	[47]
Weimaraner	75.40				0.66	[45]
West Highland White Terrier	2136.00			0.29		[12]
Whippet	87.00	0.035		0.29		[12]
	56.43				0.88	[45]
