# Supplementary material for: Comparative Analysis of Genome Diversity in Bullmastiff Dogs
Source: PLoS One. 2016 Jan 29;11(1):e0147941. doi: 10.1371/journal.pone.0147941 (PMC4732815; doi:10.1371/journal.pone.0147941)
Supplement: S2 Table — Values previously published in [12]. (PDF) [file pone.0147941.s005.pdf]

S2 Table

**Breed**	**Mean inbreeding coefficient (*F*)**
Barbet	-0.028
Beagle	0.041
Bearded Collie	-0.033
Border Collie	0.082
Braque Saint-Germain	-0.004
Bull Terrier	-0.101
Bulldog	0.017
Bullmastiff	0.035
Cavalier King Charles Spaniel	0.041
Dalmatian	-0.008
Dobermann	-0.054
English Setter	0.027
German shepherd dog	0.043
Golden retriever	0.002
Great Dane	0.046
Irish Red And White Setter	0.069
King Charles Spaniel	-0.053
Labrador retriever	0.021
Leonberger	-0.041
Romagna Water Dog	-0.021
Rottweiler	0.005
Smooth-haired Dachshund	0.092
West Highland White Terrier	-0.005
Whippet	0.125
