# Supplementary material for: Comparative Analysis of Genome Diversity in Bullmastiff Dogs
Source: PLoS One. 2016 Jan 29;11(1):e0147941. doi: 10.1371/journal.pone.0147941 (PMC4732815; doi:10.1371/journal.pone.0147941)
Supplement: S3 Table — (PDF) [file pone.0147941.s006.pdf]

S3 Table

Breed	Length (Mb)	Proportion of genome (%)	No. of runs	No. of SNPs per run	F_{ROH}
Bernese Mountain Dog (n=12)	>1	10.10	130.58	89.19	0.104
	>2	4.82	40.83	137.08	0.050
	>4	1.02	4.92	252.03	0.011
	>8	0.03	0.08	420.00	0.000
Border Terrier (n=25)	>1	12.89	160.76	94.49	0.134
	>2	6.59	55.08	143.24	0.069
	>4	1.28	6.00	263.03	0.014
	>8	0.04	0.12	461.33	0.000
Bullmastiff (n=12)	>1	14.10	170.49	99.29	0.147
	>2	7.73	63.32	148.09	0.081
	>4	1.81	8.42	273.03	0.019
	>8	0.19	0.43	564.48	0.002
Cocker Spaniel (n=14)	>1	13.46	149.93	112.23	0.137
	>2	8.12	60.36	169.81	0.084
	>4	2.93	13.29	285.38	0.031
	>8	0.33	0.86	500.50	0.004
Doberman Pinscher (n=25)	>1	13.12	163.28	92.62	0.136
	>2	6.55	52.96	143.48	0.069
	>4	1.72	8.12	254.35	0.018
	>8	0.06	0.16	356.75	0.001
English Bulldog (n=13)	>1	14.69	188.38	93.56	0.151
	>2	6.99	57.46	147.04	0.073
	>4	1.50	7.08	265.24	0.016
	>8	0.17	0.46	510.33	0.002
Greyhound (n=11)	>1	12.97	156.64	99.39	0.135
	>2	6.95	54.45	154.67	0.072
	>4	2.16	10.18	261.61	0.023
	>8	0.13	0.36	402.75	0.001
Jack Russell Terrier (n=12)	>1	6.08	65.42	123.61	0.061
	>2	3.87	26.75	192.54	0.039
	>4	1.66	6.58	343.78	0.017
	>8	0.56	1.33	583.81	0.006
Labrador Retriever (n=14)	>1	12.90	137.29	121.28	0.133
	>2	8.28	59.00	182.33	0.087
	>4	3.27	14.57	297.15	0.035
	>8	0.40	1.07	522.87	0.004
Nova Scotia Duck Trolling Retriever (n=23)	>1	12.98	146.48	108.21	0.135
	>2	7.92	59.78	162.77	0.083
	>4	2.63	12.22	270.73	0.028
	>8	0.19	0.52	468.33	0.002
Rottweiler (n=12)	>1	11.35	142.17	93.40	0.118
	>2	5.68	47.58	140.73	0.060
	>4	1.09	5.25	251.89	0.011
	>8	0.06	0.17	515.00	0.001
Standard Poodle (n=12)	>1	12.21	130.75	121.03	0.125
	>2	7.91	57.00	181.64	0.082
	>4	3.02	13.17	303.57	0.032
	>8	0.43	1.17	515.50	0.005
Weimaraner (n=26)	>1	13.09	157.19	96.91	0.136
	>2	6.91	52.12	151.47	0.073
	>4	2.26	10.65	250.63	0.024
	>8	0.08	0.19	482.80	0.001
