# Supplementary material for: Comparative Analysis of Genome Diversity in Bullmastiff Dogs
Source: PLoS One. 2016 Jan 29;11(1):e0147941. doi: 10.1371/journal.pone.0147941 (PMC4732815; doi:10.1371/journal.pone.0147941)
Supplement: S4 Table — (PDF) [file pone.0147941.s007.pdf]

S4 Table

**Breed**	**MLH**
Bernese Mountain Dog (n=12)	0.205
Border Terrier (n=25)	0.201
Bullmastiff (n=12)	0.200
Cocker Spaniel (n=14)	0.229
Doberman Pinscher (n=25)	0.179
English Bulldog (n=13)	0.188
Greyhound (n=11)	0.197
Jack Russell Terrier (n=12)	0.286
Labrador Retriever (n=14)	0.249
Nova Scotia Duck Tolling Retriever (n=23)	0.232
Rottweiler (n=12)	0.210
Standard Poodle (n=12)	0.273
Weimaraner (n=26)	0.195
