# Supplementary material for: Comparative Analysis of Genome Diversity in Bullmastiff Dogs
Source: PLoS One. 2016 Jan 29;11(1):e0147941. doi: 10.1371/journal.pone.0147941 (PMC4732815; doi:10.1371/journal.pone.0147941)
Supplement: S5 Table — (PDF) [file pone.0147941.s008.pdf]

S5 Table

Cluster	Average Relationship Coefficient
1	0.29
2	0.16
3	0.13
4	0.19
5	0.19
6	0.21
7	0.20
8	0.16
9	0.12
10	0.16
11	0.19
